# Ten simple rules for writing a *PLOS Computational Biology* quick tips article

**DOI:** 10.1371/journal.pcbi.1011689

**Published:** 2023-12-21

**Authors:** Patricia M. Palagi, Russell Schwartz, Scott Markel, B. F. Francis Ouellette

**Affiliations:** 1 SIB Swiss Institute of Bioinformatics, Lausanne, Switzerland; 2 Carnegie Mellon University, Pittsburgh, Pennsylvania, United States of America; 3 Dassault Systemes BIOVIA, San Diego, California, United States of America; 4 Bioinformatics.ca, Montréal, Quebec, Canada; Johns Hopkins University, UNITED STATES

## Introduction

Seventeen years have passed since Phil Bourne inaugurated the Ten Simple Rule (TSR) collection in *PLOS Computational Biology* (PLOS CB) with a paper entitled “Ten Simple Rules for Getting Published” [1]. At that time, the change in how we communicate sciences had already begun [2]: social media was blooming, the information deluge was ongoing, and the fear of missing information was anchored in each of us. The Ten Simple Rules collection filled a space in scientific publications where researchers eager to share their experiences, wisdom, and doubts could quickly do it using a colloquial narrative. The topics covered were broad themes in scientific practice, such as soft skills and career development, captured in a concise and quick-to-read format, something between a blog post and a scientific article.

This type of article attracted much interest from readers. In 2018, the collection reached the milestone of 1,000 Rules [3], and today this figure is above 250 papers (2,500 Rules!) [4]. With the increase of technical and scientific topics, in 2013, *PLOS Computational Biology* tried a new experience with a similar format—we introduced “Quick Tips” (QT) articles—with the attempt to make a clear distinction between the more specific and focused scientific activities and skills presented with resources, databases, and other tools in Quick Tips versus the broader themes presented in a Ten Simple Rules article.

“Ten Simple Rules for Writing a PLOS Ten Simple Rules Article” [5] explains the Ten Simple Rules concept, format, and reasoning very well and is still relevant today. Inspired by that article, we wrote this Ten Simple Rules paper intending to accomplish a similar task: explaining to our community what a Quick Tips article is about and how a Quick Tips article differs from a Ten Simple Rules article. The authors are the Section Editors of the Education Collection (PP and BFFO), which encompasses the Quick Tips, and the Section Editors of the Ten Simple Rules Collection (RS and SM). In our work at PLOS CB, we are routinely deliberating the merits of a submission being a Ten Simple Rules or a Quick Tips, and for this reason, we decided to put these Ten Simple Rules about Quick Tips together. Historically, several papers were submitted as Ten Simple Rules that should have been Quick Tips. Still, we will refrain from commiserating about things not done but rather present what we hope will be a clear distinction that will make it obvious in the future why articles are best considered as Quick Tips versus Ten Simple Rules (or vice-versa). We hope and plan for this present article to be helpful for future writers willing to contribute to this collection and to help clarify the differences between Ten Simple Rules and Quick Tips.

**Fig 1:**
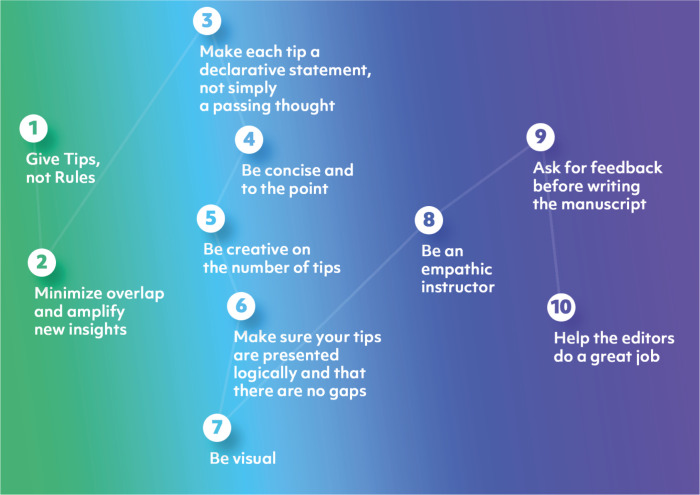
Summary of the Ten Simple Rules presented in this manuscript.

Why is this not a Quick Tips article but a Ten Simple Rules article, and why are we not as endearing as Dashnow, Lonsdale, and Bourne were in doing a “Ten Quick Tips for Writing a PLOS Quick Tips Article”? The reason is simple. As stated in Rule 1, this is a soft-skills article: it aims to explain how best to write a particular type of manuscript for PLOS. Thus, it is not a Quick Tips but a Ten Simple Rules article. In Fig 1, we summarize the Ten Simple Rules we are presenting.

### Rule 1: Give tips, not rules

Quick Tips are for guiding readers on developing scientific and technical skills on using databases, resources, computational or data analysis methods and tools. Quick Tips can teach how to analyze network data [6], use the Gene Ontology (GO) [7], or use deep learning in biology [8]. They have a specific educational perspective. With a Quick Tips article, you drive the reader towards learning and applying what they are learning. They are quick tips, not simple rules. As such, each tip needs to inform the reader about a different aspect of the overall problem (computational) biologists want to get better at.

Ten Simple Rules are more to develop soft skills, career promotion/advancement (how to organize a scientific conference [9], write your cover letter [10], write a literature review [11], etc.), or other broad issues in scientific practice (e.g., models for successful collaboration [12,13], developing scientific communities [14], or practicing inclusive science [15,16]).

### Rule 2: Minimize overlap and amplify new insights

When putting together a Quick Tips article, ensure there is no redundancy with other Quick Tips or other educational articles. The Quick Tips articles have yet to become as famous as its sister collection, Ten Simple Rules, which counts 255 articles so far [4]. Still, plenty of valuable Quick Tips and education articles in PLOS and other journals may have treated the same topic as yours. But if you think you have a different or unique perspective on the same topic, do not forget to reference all those relevant to your Quick Tips in your manuscript and explain what makes yours unique. Paraphrasing Dashnow, Lonsdale, and Bourne’s wise advice in Rules 7 and 9: summarize, cite, and write well [5].

### Rule 3: Make each tip a declarative statement, not simply a passing thought

The title of the tip is essential, and it needs to state a tip. At a glance, the reader must understand your main key message, which must simultaneously be persuasive and noteworthy.

When you declare a tip, make sure it is a statement and to the point. You are providing a unit of advice to your readers to perform (or not) specific tasks, and as such, you want to avoid misinterpretations. By making your tips declarative statements, you can ensure they are unambiguous and leave no room for interpretation or confusion. If you provide an opinion, provide evidence to sustain your point of view and convince your readers of your argument.

### Rule 4: Be concise and to the point

Less is more. Quick Tips papers are currently part of the *PLOS Computational Biology* Education collection [17,18], and as for any article in this collection, a Quick Tips article is about 2,000 to 2,500 words [19]. From a pedagogical perspective, some educational models support the value of being concise (e.g., Miller) [20], which we support in the PLOS Education collection. However, there is some flexibility here. The story of your manuscript may be more important than the length of the manuscript you are submitting. Word limits provide boundaries and are a great tool to make you reflect on the primary key messages you want to deliver. You cannot neglect good writing practices because Quick Tips papers are short. Make sure to read and apply the excellent rules provided by Ehrhart and Evelo in [21] to your manuscript.

### Rule 5: Be creative on the number of tips

**Fig 2:**
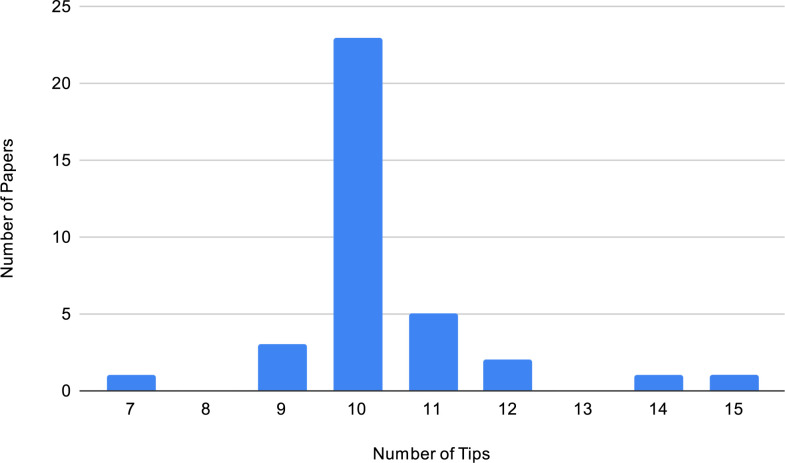
Distribution of the number of tips presented in the 36 Quick Tips articles from 2020–09 to 2023–10 [22].

Another important difference between the Quick Tips articles and Ten Simple Rules articles is that you are not restricted to 10 tips, as opposed to Ten Simple Rules, where 10 is one of the rules [5]. Please feel free to do so if it makes sense to have 8, 9, or 13 tips. As we see in Fig 2, the number of tips we have seen in Quick Tips articles ranges from 7 to 15.

### Rule 6: Make sure your tips are presented logically and that there are no gaps

Quick Tips are like recipes, like a procedural order of execution of tasks for a given topic. They should follow at least a logical order. Consider them a “sorted checklist” that your readers will follow and apply. As for a recipe, you can’t miss any ingredients or procedures, as the result may not be as expected.

### Rule 7: Be visual

Using images in a manuscript can help to make the content more accessible, engaging, and visually appealing to readers, and as the adage says: “an image is worth a thousand words.” Images can help visualize or clarify a concept or idea you are discussing in the text and provide evidence for your claims in the tips. As Weinstein and Sumeracki, two cognitive scientists, say “Pictures are usually remembered better than words, and can provide additional memory cues” and thus help learning and understanding [23]. Thus, we recommend Quick Tips authors incorporate a figure summarizing and illustrating their tips (Fig 1). By the way, we recommend the same for Ten Simple Rules authors.

Alternatively, suppose you do not use a figure. In that case, we recommend including a table in the introduction that declares the tips in a summarized way and allows the reader to get an overview of the text to follow.

For all images presented on the web or within a publication, it is important to have some alternative text (Alt-Txt) for visually impaired readers [24,25] and also to consider a color palette that color blind people will be able to interpret [26].

### Rule 8: Be an empathic instructor

Quick Tips are educational articles and thus serve to teach scientific or technical skills to your readers. Be mindful of your language (avoid jargon, define terms), and be specific, clear, and concise. Quick Tips need to be written as a tutorial used by a classroom teacher or a reader to learn from you. Your ambition: be the reference article about teaching that topic.

You can write a Quick Tips article alone, but collaborating is better. Seven of the 10 most cited PLOS CB Quick Tips articles have three or more authors [22]. Your tips should be messages from a community of instructors you represent for a community of readers. When you write a Quick Tips article, you (singular, but hopefully plural) speak as experts with an aura of knowledge, authority, and empathy for your readers.

### Rule 9: Ask for feedback before writing the manuscript

Well ahead of starting to work on that manuscript, outline the tips, and send this together with a summary to ploscompbiol@plos.org. We will happily comment and guide you before you spend too much time writing a full manuscript. One common question where people still need clarification: should it be a Ten Simple Rules or a Quick Tips submission? If you are unsure after reading this publication, please contact the section editors or *PLOS Computational Biology* directly (ploscompbiol@plos.org). As always, it is also a good idea to consult and share with colleagues before you submit any article.

### Rule 10: Help the editors do a great job

As for all *PLOS Computational Biology* articles, we send the submissions for peer review, so don’t forget to include many suggestions for potential reviewers (5 or 6 is ideal) from which the section editors may select. Identify experts within your field who can serve as independent, objective reviewers and are free from competing interests. At the same time, since Quick Tips are for educational purposes and have a particular format (not to be confused with usual research papers), these experts should be aware of the Quick Tips format. They should also have an open and teaching mindset. Remember that suggesting recent collaborators or other researchers at your institution is inappropriate, as it would be for any other manuscript submission.

## Conclusions

Ten Simple Rules and Quick Tips are fundamentally similar types of articles. Except for Rules 1 and 5, all the other rules in this manuscript can also apply to the Ten Simple Rules papers. If we were to give you extra and particular advice, it would be: never underestimate Rule 2. Minimize overlap and amplify new insights.

Ten Simple Rules, Quick Tips, and the Education collections already have plenty of exciting and insightful manuscripts, which are excellent resources for teaching and learning. But there is always a place for your preferred topic; we welcome and look forward to your new ideas. We endeavor to get back to you in a timely way.

As a way to say “thank you,” we want to remind new potential authors of the Quick Tips and Ten Simple Rules articles that these articles (which also include all in the PLOS CB Education collection) are what is referred to as “Front Matter,” and PLOS has no publication charges (no APC) on Front Matter papers. Like all PLOS articles, Quick Tips articles are fully Open Access (Gold) and are free to you as the author (Open Access Diamond). Front matter is like the author and PLOS giving to the community, so there are no charges.
